# Life-threatening giant esophageal neurofibroma with severe tracheal stenosis: a case report

**DOI:** 10.1186/s40792-018-0517-1

**Published:** 2018-09-03

**Authors:** Eisuke Booka, Mitsuhide Kitano, Yutaka Nakano, Koki Mihara, Shin Nishiya, Ryo Nishiyama, Shintaro Shibutani, Tomohisa Egawa, Atsushi Nagashima

**Affiliations:** 1 Department of Surgery, Saiseikai Yokohamashi Tobu Hospital, 3-6-1 Shimosueyoshi, Tsurumi-ku, Yokohama, Kanagawa 230-0012 Japan; 2 Department of Trauma and Emergency Surgery, Saiseikai Yokohamashi Tobu Hospital, 3-6-1 Shimosueyoshi, Tsurumi-ku, Yokohama, Kanagawa 230-0012 Japan; 3 Department of Surgery, Saiseikai Kanagawaken Hospital, 6-6 Tomiya-cho, Kanagawa-ku, Yokohama, Kanagawa 221-8601 Japan

**Keywords:** Esophagus, Neurofibroma, Extracorporeal membrane oxygenation (ECMO), S-100 protein, Immunohistochemical staining

## Abstract

**Background:**

Benign esophageal tumors are relatively rare, and a neurofibroma in the esophagus is extremely rare. Dysphagia is the most common clinical manifestation in patients with esophageal neurofibroma, and no cases of giant esophageal neurofibroma with severe tracheal stenosis have been reported.

**Case presentation:**

A 73-year-old woman presented with shortness of breath, and computed tomography scan exhibited a giant mediastinal tumor causing severe tracheal stenosis. An upper gastrointestinal endoscopy revealed a giant submucosal lesion without mucosal changes located 18–23 cm from the incisor teeth. ^18^F-fluorodeoxyglucose (FDG)-positron emission tomography image revealed an upper mediastinal homogeneous mass and left supraclavicular lymph node with increased FDG accumulation. We performed endoscopic ultrasound-guided fine-needle aspiration biopsy; however, a definitive diagnosis could not be determined. During further investigation, her shortness of breath suddenly worsened and she suffered from wheezing. Because of risk of smothering, we decided to perform quasi-urgent lifesaving surgery. Under the preparation of extracorporeal membrane oxygenation (ECMO) when tracheal intubation fails, bronchial blocker was inserted over the tracheal stenosis and the left-lung ventilation was performed via intubation alone. Under general anesthesia, the patient was placed in the left lateral position and we performed right thoracotomy. The tumor strongly adhered to the trachea; however, the trachea or recurrent laryngeal nerves were not damaged in the surgery. Following esophagectomy, we performed gastric conduit reconstruction through the posterior mediastinum, and hand-sewn anastomosis was performed in the left neck. Immunohistochemical staining was positive for S-100 but negative for c-KIT, CD34, α-SMA, and desmin; these morphological and immunohistochemical characteristics were consistent with the diagnosis of neurofibroma.

**Conclusions:**

It is often difficult to diagnose esophageal neurofibroma preoperatively. The preparation of ECMO could be considered in patients with severe airway obstruction for safe tracheal intubation. This is the first case of life-threatening giant esophageal neurofibroma with severe tracheal stenosis.

## Background

Benign esophageal tumors are relatively rare, and leiomyoma accounts for 80% of these tumors; however, they are extremely difficult to diagnose preoperatively [1]. A definitive diagnosis requires histological confirmation and immunohistochemical staining. Neurofibromas are rare, and dysphagia is the most common clinical manifestation in patients with esophageal neurofibroma [2]. Here, we report the first case of life-threatening giant esophageal neurofibroma with severe tracheal stenosis.

## Case presentation

**Fig. 1:**
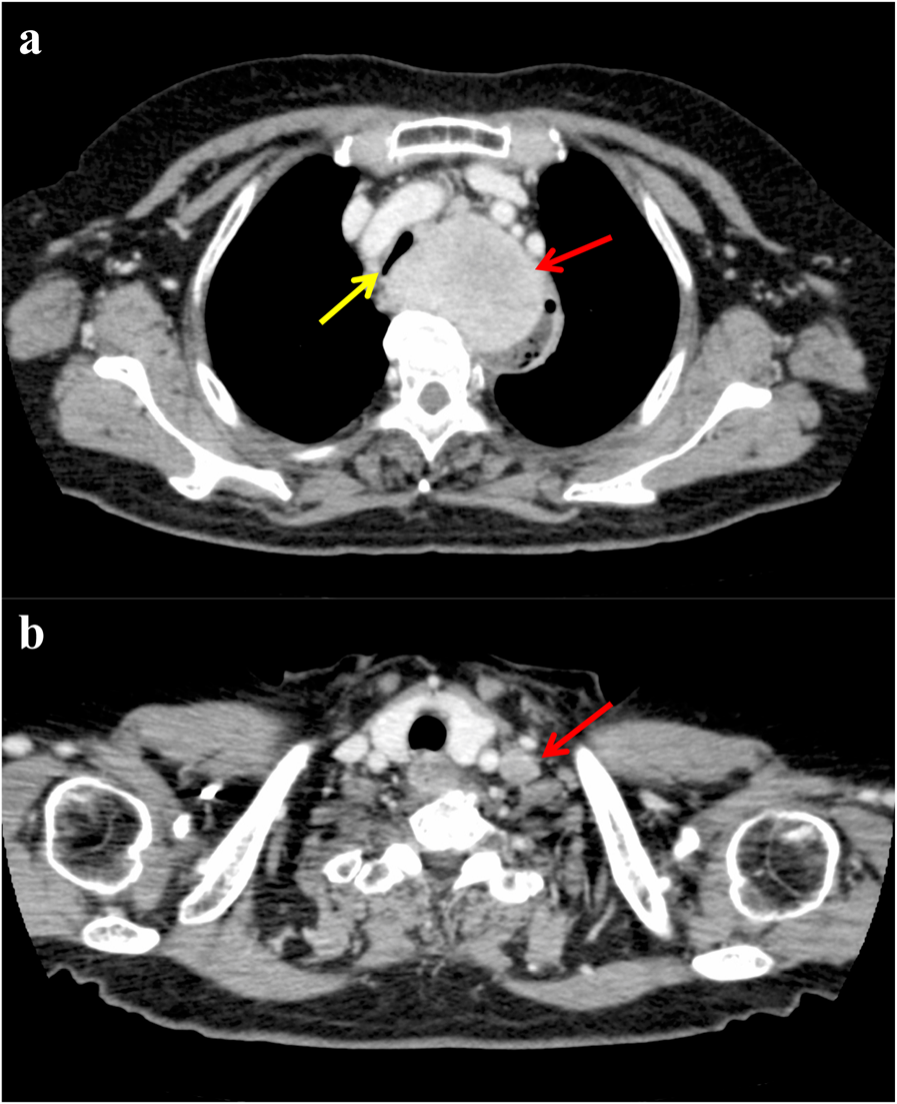
Computed tomography imaging scan. **a** Red arrow indicates the mediastinal tumor and yellow arrow indicates tracheal stenosis. **b** Red arrow indicates supraclavicular lymph node enlargement

**Fig. 2:**
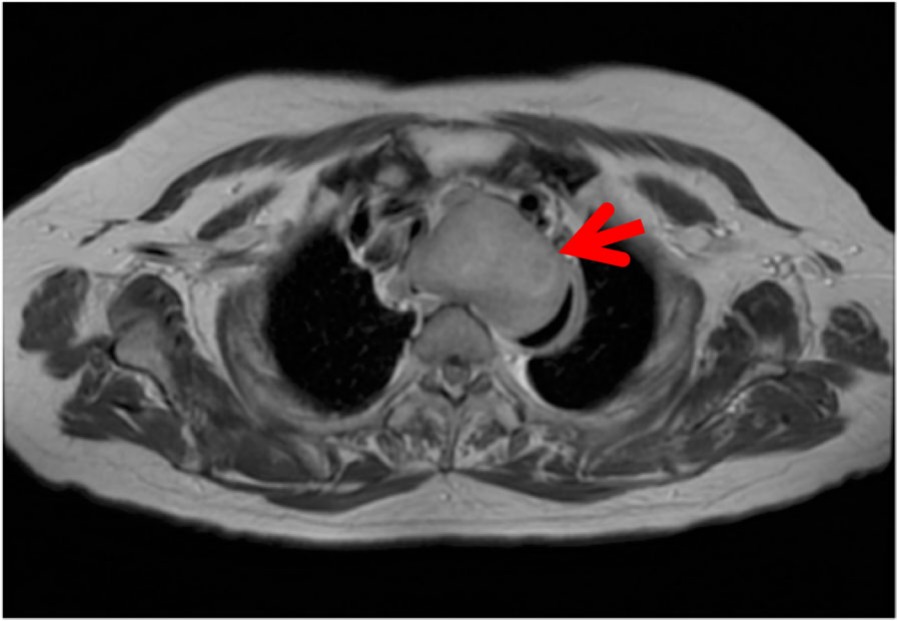
T2WI magnetic resonance imaging revealed a heterogeneous inner structure

**Fig. 3:**
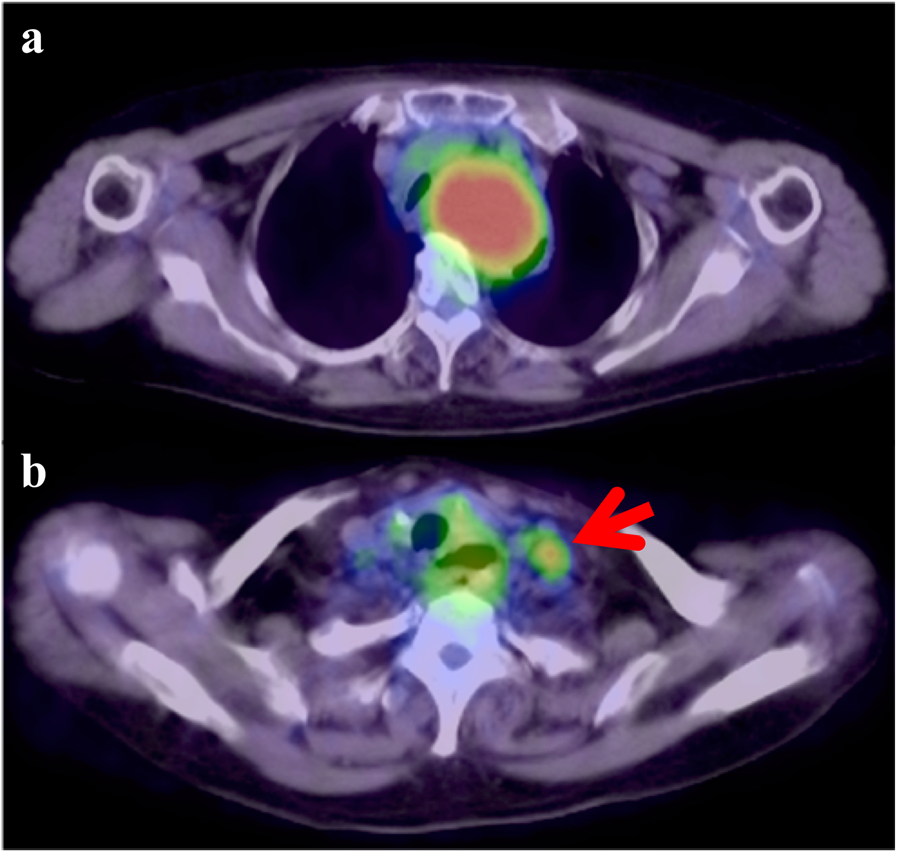
^18^F-fluorodeoxyglucose (FDG)-positron emission tomography image revealed an upper mediastinal homogeneous mass (**a**) and left supraclavicular lymph node with increased FDG accumulation (**b**)

A 73-year-old woman presented with shortness of breath; however, the symptom was not serious. A computed tomography scan was performed at an outpatient clinic in her neighborhood, which revealed a giant mediastinal tumor (Fig. 1a) and enlarged left supraclavicular lymph node (Fig. 1b). At first, not an esophageal tumor but malignant lymphoma was suspected, and she was referred to another hospital specializing in blood cancers. At the second hospital, an upper gastrointestinal endoscopy was performed showing a giant submucosal lesion without mucosal changes located 18–23 cm from the incisor teeth. Endoscopic ultrasonography (EUS) revealed a homogeneous and hypoechoic solid lesion with a clear margin appearing to originate from the esophageal submucosa. Magnetic resonance imaging clearly showed a solid mass 6 cm in diameter that was compressing her trachea and esophagus (Fig. 2). ^18^F-fluorodeoxyglucose (FDG)-positron emission tomography imaging revealed an upper mediastinal homogenous mass and left supraclavicular lymph node with increased FDG accumulation (Fig. 3a, b). The standardized uptake value of the upper mediastinal lesion was 9.4, suggesting high glycolytic activity in the mass. Laboratory data were unremarkable with normal levels of serum tumor markers such as CEA, AFP, and CA 19-9. The level of interleukin-2 receptor was within normal limits, and CRP remained negative. There were no pigmented patches, and von Recklinghausen disease (VRD) was not diagnosed.

EUS-guided fine-needle aspiration (FNA) biopsy was conducted in order to provide a definitive diagnosis. Three EUS-FNA specimens revealed spindle cell tumors; however, a definitive diagnosis was not determined with immunohistochemical staining. Immunohistochemical staining was negative for c-KIT, CD34, cytokeratin AE1/AE3, p53, and desmin, but a partial positive for S-100. The patient was required to undergo surgery with general anesthesia for further investigation or treatment; however, tracheal intubation was considered to be a very high risk at the second hospital due to the severe tracheal stenosis. Hence, she was referred to our hospital for further treatment after 5 months of visiting the second hospital.

At our hospital, we reviewed previous findings, and the differential diagnoses were leiomyoma, gastrointestinal stromal tumor (GIST), and neurogenic tumor. Although EUS-FNA specimens revealed spindle cell tumors, malignant lymphoma was not excluded completely because of left supraclavicular lymph node with increased FDG accumulation. A definite diagnosis could be reached by performing left supraclavicular lymph node dissection under general anesthesia if the giant tumor was malignant lymphoma. However, tracheal intubation could be impossible because of severe tracheal stenosis, and extracorporeal membrane oxygenation (ECMO) is considered to be life-saving when tracheal intubation fails. The catheters were inserted into internal jugular vein and femoral vein before inducing anesthesia. Under the preparation of VV-ECMO when tracheal intubation fails, single-lumen tracheal tube was inserted over the tracheal stenosis, and the ventilation was performed via intubation alone. Under general anesthesia, left supraclavicular lymph node was dissected; however, the dissected lymph node revealed only inflammation and malignant lymphoma was excluded completely. Her symptom of shortness of breath was not so serious although severe tracheal stenosis was observed; hence, there was no urgency of treatment. However, her shortness of breath suddenly worsened after a few days of lymph node dissection and she suffered from wheezing. We decided to perform quasi-urgent lifesaving surgery. Preoperative diagnosis was GIST because of spindle cell and increased FDG accumulation, and differential diagnoses were leiomyoma and neurogenic tumor. Because the tumor was too large to perform enucleation, esophagectomy was first considered.

**Fig. 4:**
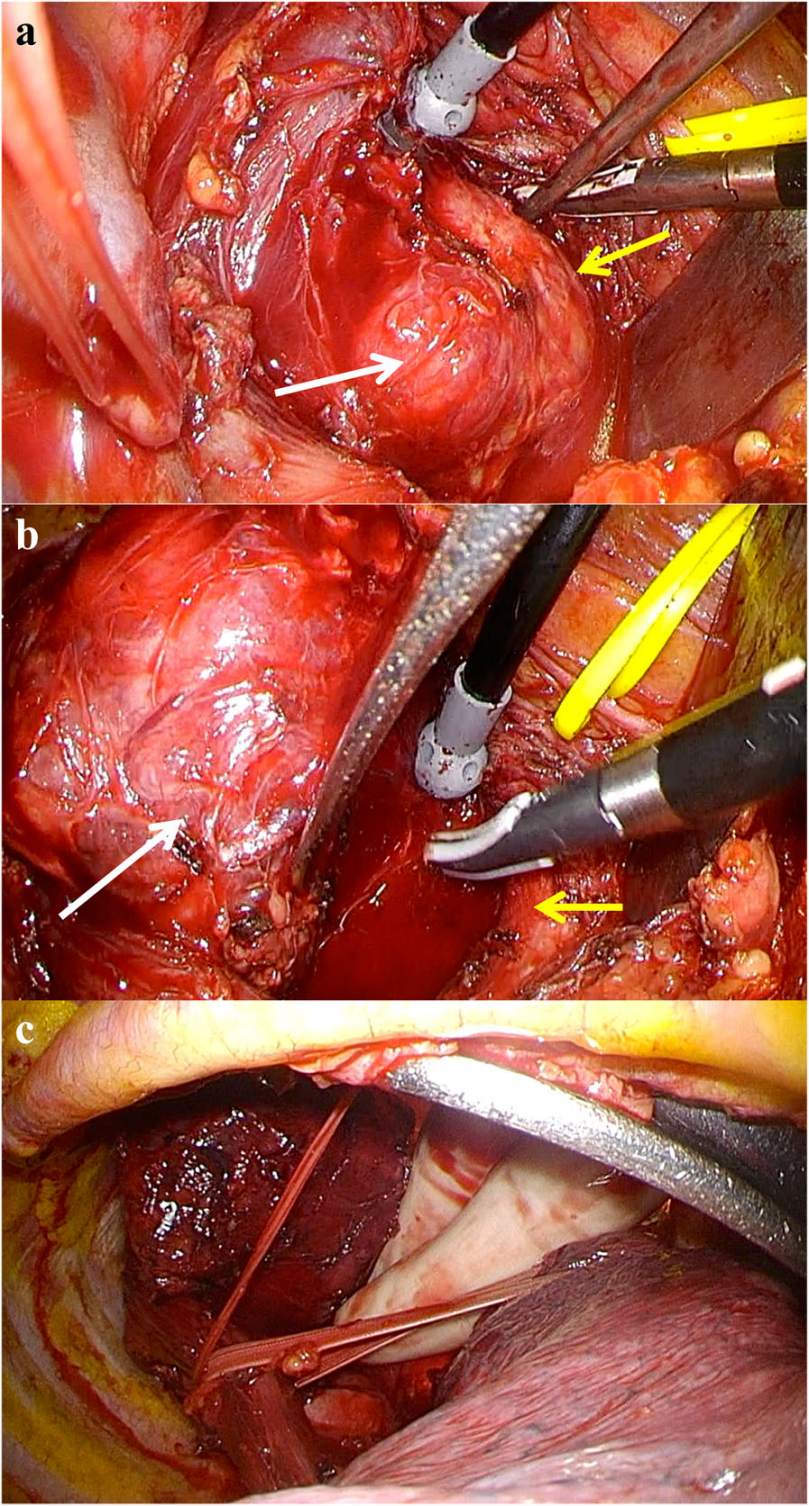
Intraoperative findings (**a**, **b**, **c**). White arrows show the mediastinal tumor and yellow arrows show the trachea (**a**, **b**)

Again, under the preparation of VV-ECMO when tracheal intubation fails, bronchial blocker was inserted over the tracheal stenosis and the left-lung ventilation was performed via intubation alone. Under general anesthesia, the patient was placed in the left lateral position, and right thoracotomy through the fourth intercostal space was performed. Tumor exploration showed the giant tumor occupying the upper mediastinum and heavily compressing her trachea (Fig. 4a). First, arch of the azygos vein was cut to expose the tumor and adjacent esophagus. The tumor strongly adhered to the trachea; however, the trachea or recurrent laryngeal nerves were not damaged in the surgery (Fig. 4b). The esophagus was encircled above and below the tumor (Fig. 4c). At that time, the tumor enucleation was considered to pose a high risk of mucosal injury or poor mucosal blood flow; thus, we decided to perform esophagectomy with tumor removal as planned. We reconstructed the esophagus using the gastric conduit through the posterior mediastinum and performed hand-sewn anastomosis at the left neck. The operation time was 565 min and blood loss was 582 ml.

**Fig. 5:**
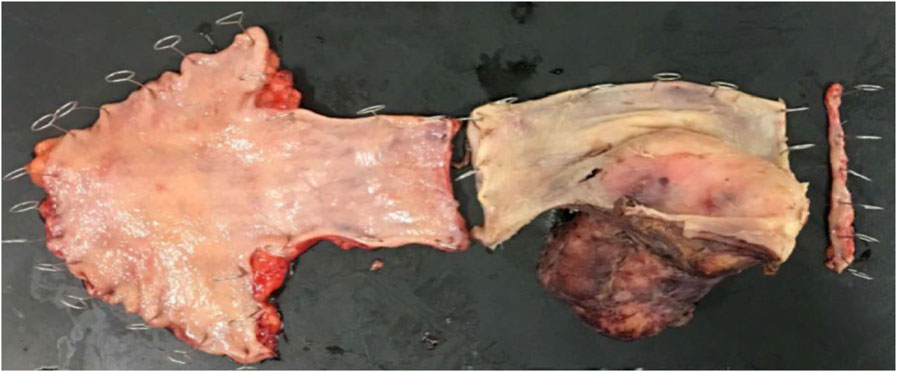
Macroscopic appearance of the tumor. The resected tumor was soft in elasticity and measured 90 × 50 × 50 mm

**Fig. 6:**
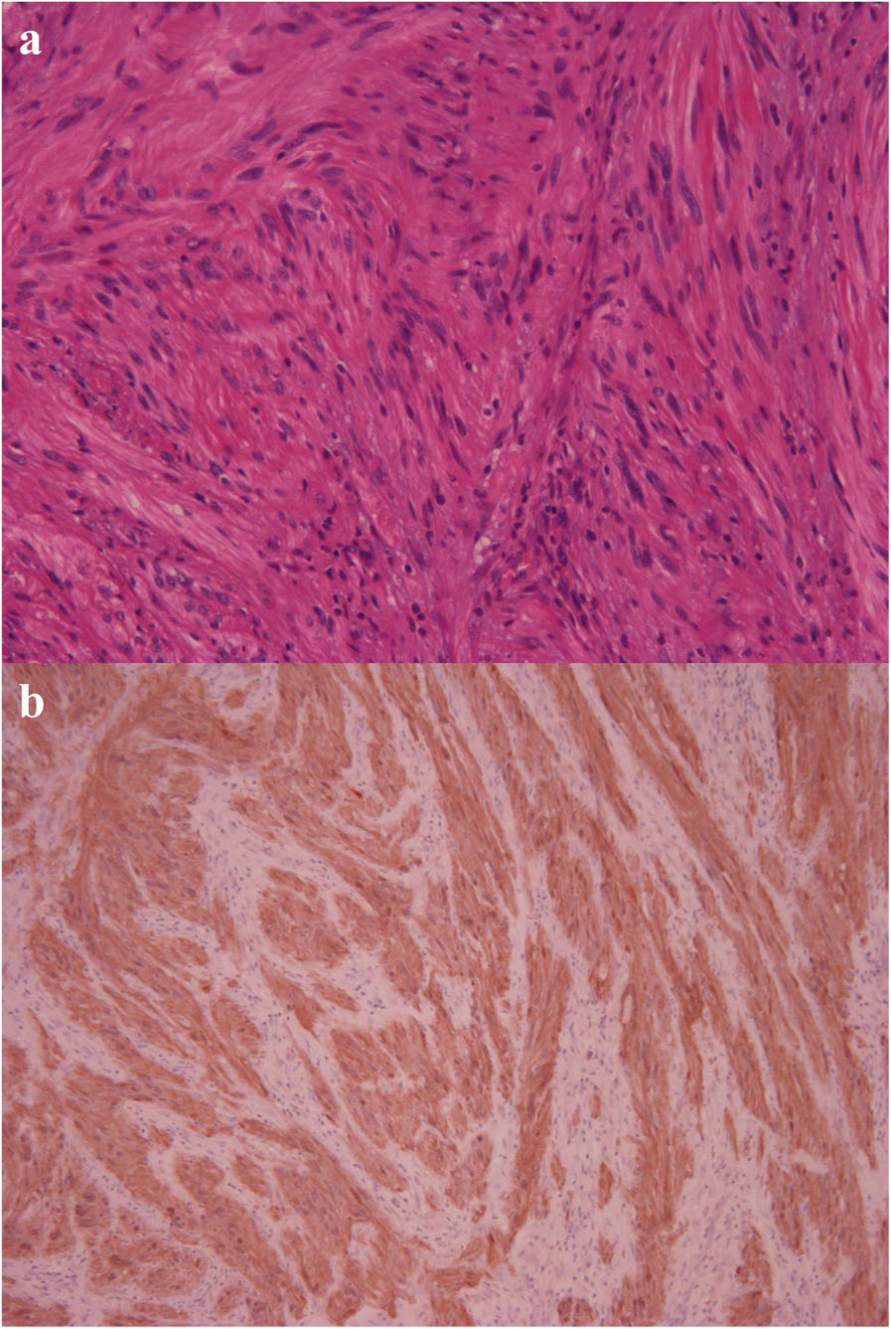
Histologic appearance of the tumor. The tumor comprised a mixture of fibrillary collagen and cords of spindle cells with nodular growth (**a**, × 50). Immunohistochemical staining results were positive for S-100 (**b**, × 80)

The resected tumor was soft in elasticity, measuring 90 × 50 × 50 mm (Fig. 5). Histologic examination revealed a large spherical nodular tumor and comprised mixed fibrillary collagen sheets and cords of spindle cells with nodular growth (Fig. 6a). No signs of atypia or significant mitotic activity were observed. Immunohistochemical staining was positive for S-100 but negative for c-KIT, CD34, α-SMA, and desmin (Fig. 6b), and these morphologic and immunohistochemical characteristics were consistent with a diagnosis of neurofibroma. Postoperatively, the patient experienced anastomotic leakage, but she was discharged on the 57th postoperative day with sufficient oral intake.

### Discussion

Benign esophageal submucosal tumors (SMT) are relatively rare, and leiomyoma is the most common, accounting for > 80% of these tumors [3]. Neurofibromas associated with esophageal SMTs are very rare, as Plachta reported neurofibromas in four cases of 432 SMTs (0.9%) [4] and Enterkine et al. reported five cases of 571 SMTs (0.9%) [5]. Neurofibromas can be associated with genetic disorders and are usually a manifestation of VRD [6]. They are typically benign tumors comprising neural and connective tissue components, such as Schwann and perineural cells as well as myofibroblasts [2]. One fourth of patients with VRD report gastrointestinal involvement [7]. There are three types of neurofibromas: localized, diffuse, and plexiform [2]. Localized neurofibromas of the gastrointestinal (GI) tract, unlike VRD, are benign nerve sheath tumors in the peripheral nervous system originating from Auerbach plexus and Meissner submucosal plexus [8, 9]. Our patient had localized neurofibromas without VRD. Localized neurofibroma is far more common in the GI tract, and to date, there has been only one report of plexiform esophageal neurofibroma associated with VRD [10].

A few reports have described localized esophageal neurofibromas, and Nishikawa et al. summarized 16 cases [2, 5, 11–16]. In addition, Yang et al. recently reported a giant esophageal neurofibroma [17]; thus, including our case, we summarized 18 cases of localized esophageal neurofibromas (Table 1). The mean age of the patients was 53.6 years (range, 26–75 years), and there was no difference in sex distribution (male:female, 8:10). Dysphasia symptoms were observed in approximately one third of patients. Tumors were located primarily in the mid to upper section of the thoracic esophagus (83%), and the mean tumor size was 6.2 cm (range, 0.5–22.5), whereas leiomyomas were located in the middle or distal regions [18].

**Table 1:** Reported cases of esophageal neurofibroma

Case	Author	Year	Age	Sex	Symptoms	Location	Size (cm)	Treatment	S-100
1	Saitoh et al. [5]	1977	26 years	M	Dysphagia	Mt	NA	NA	NA
2	Goto et al. [5]	1982	56 years	F	Abnormal shadow in esophagus	Lt	NA	NA	NA
3	Oguchi et al. [5]	1983	55 years	M	Prolapse of tumor	Ce	22.5 × 4.5	Enucleation	NA
4	Inoue et al. [5]	1984	50 years	M	Abnormal shadow in esophagus	Mt	0.7 × 0.5	Enucleation	NA
5	Hishikawa et al. [12]	1984	55 years	M	Epigastric pain	Mt	2.0 × 2.0	Enucleation	NA
6	Saitoh et al. [16]	1985	64 years	F	Abnormal shadow in esophagus	Mt	4.2 × 4.0	enucleation	Positive
7	Fujiwara et al. [5]	1985	75 years	F	Intestinal bleeding	NA	NA	NA	NA
8	Madrid et al. [15]	1986	53 years	F	Dysphagia	Ut	8.0 × 6.0	Esophagectomy	NA
9	Hara et al. [5]	1987	67 years	F	Dysphagia	Mt	1.7 × 1.5	Enucleation	Positive
10	Sugiyama et al. [5]	1989	36 years	M	Abnormal shadow in esophagus	Ut	11.0 × 6.5	Esophagectomy	NA
11	Ohashi et al. [5]	1990	34 years	M	Abnormal shadow in esophagus	Ut	3.0 × 2.7	Enucleation	Positive
12	Ramirez et al. [11]	1992	61 years	F	NA	Mt	NA	NA	NA
13	Fujita et al. [5]	1993	48 years	F	Abnormal shadow in esophagus	Lt	6.0 × 5.0	Esophagectomy	Positive
14	Lee et al. [14]	1997	58 years	F	Dysphagia and odynophagia	Ut	4.0 × 6.0	Enucleation	Positive
15	Ishii et al. [13]	2002	35 years	F	Sensation of foreign body in the hypopharynx	Ce	NA	Enucleation	NA
16	Nishikawa et al. [2]	2013	56 years	F	Epigastric discomfort	Mt	3.4 × 2.8	Enucleation	Positive
17	Yang et al. [17]	2017	63 years	M	Dysphagia, belching, and retrosternal pain	Ut	12.0 × 3.0	Enucleation	Negative
18	Present case		73 years	F	Shortness of breath	Ut	9.0 × 5.0	Esophagectomy	Positive

S-100; postoperative immunohistochemical staining for S-100 protein, *NA* not available data

In summary, dysphagia most commonly manifests in patients with esophageal neurofibromas. Our case is the first report of a patient suffering from wheezing with severe tracheal stenosis and requiring quasi-urgent surgery. The utility of ECMO in patients with severe airway obstruction was reported in the systematic review [19]. From the systematic review, all cases reported a favorable outcome with all patients surviving to hospital discharge without significant complications [19]. In our case, under the preparation of VV-ECMO when tracheal intubation fails, we were able to perform tracheal intubation safely and radical surgery thereafter.

It is often difficult to diagnose localized esophageal neurofibroma using diagnostic imaging; thus, histopathologic diagnosis of neurofibroma is necessary in most cases. We hesitated to perform radical surgery without a definite diagnosis as tumor resection and separation from the trachea seemed to be difficult. If the giant tumor was malignant lymphoma, chemotherapy would be effective, and the patient would be saved without radical surgery. It is well known that malignant tumor or malignant lymphoma had increased FDG accumulation; however, Watanabe et al. reported benign esophageal SMT such as esophageal schwannoma had also increased FDG accumulation [20]. The histopathologic characteristic appearance of neurofibroma consists of spindle-shaped cells associated with collagen fibrils. Additionally, immunohistochemical staining aids in distinguishing neurogenic from myogenic and other submucosal tumors. In our case, the tumor comprised fibrillary collagen and spindle cells organized in whorls, and it was positive for S-100 but negative for c-KIT, CD34, α-SMA, and desmin.

Surgery is the primary treatment for esophageal neurofibroma if clinical symptoms are present. Enucleation of esophageal neurofibromas is sufficient treatment if the tumor is small, and the thoracoscopic approach could be adapted for such small tumors [2, 21–23]. However, in our case, a definitive diagnosis could not be determined for giant esophageal SMT; malignancy was suspected due to increased FDG accumulation, and performing right thoracic esophagectomy was necessary.

## Conclusions

It is often difficult to diagnose esophageal neurofibroma preoperatively. The preparation of ECMO could be considered in patients with severe airway obstruction for safe tracheal intubation. This is the first case of life-threatening giant esophageal neurofibroma with severe tracheal stenosis.

## Abbreviations

ECMO: Extracorporeal membrane oxygenation
EUS: Endoscopic ultrasonography
FDG: Fluorodeoxyglucose
FNA: Fine-needle aspiration
GI: Gastrointestinal
GIST: Gastrointestinal stromal tumor
SMT: Submucosal tumor
VRD: von Recklinghausen disease
